# Biomechanics of Interspinous Devices

**DOI:** 10.1155/2014/839325

**Published:** 2014-07-09

**Authors:** Paolo D. Parchi, Gisberto Evangelisti, Antonella Vertuccio, Nicola Piolanti, Lorenzo Andreani, Valentina Cervi, Christian Giannetti, Giuseppe Calvosa, Michele Lisanti

**Affiliations:** ^1^1st Orthopedic Division, University of Pisa, Via Paradisa 2, 56125 Pisa, Italy; ^2^Orthopedic Division, S. Maria Maddalena Hospital, Borgo S. Lazzero, 56048 Volterra, Italy

## Abstract

A number of interspinous devices (ISD) have been introduced in the lumbar spine implant market. Unfortunately, the use of these devices often is not associated with real comprehension of their biomechanical role. The aim of this paper is to review the biomechanical studies about interspinous devices available in the literature to allow the reader a better comprehension of the effects of these devices on the treated segment and on the adjacent segments of the spine. For this reason, our analysis will be limited to the interspinous devices that have biomechanical studies published in the literature.

## 1. Introduction

A number of interspinous devices (ISD) have been introduced in the lumbar spine implant market. Designs vary from static spacers to dynamizing spring-like devices and they are composed of an array of different materials including titanium, polyetheretherketone (PEEK), and elastomeric compounds [1].

The main indications for their use include lumbar canal stenosis, Grade I degenerative spondylolisthesis, discogenic low back pain, nontraumatic instability, lumbar disc herniation, and facet syndrome [2, 3]. The aims are to unload the facet joints, to restore foraminal height, and to provide sufficient stability especially in extension but still allow motion in the treated segment [4]. Motion preserving interspinous implants allow the preservation of a range of motion (ROM) in the implanted segment, thus avoiding or limiting possible overloading and early degeneration of the adjacent segments as induced by fusion [5]. A different class of interspinous devices, recently introduced in the market, is instead used to obtain the fusion of interspinous space [6].

The interspinous devices often can be implanted in lumbar spine using a minimal or mini-invasive approach in local anesthesia. This is one of the main reasons that has led to a boom of the use of interspinous devices in the last decade for a wide range of lumbar pathologies. Unfortunately, the use of the interspinous devices often is not associated with real comprehension of the biomechanical role of these devices.

The aim of this paper is to review the biomechanical studies about interspinous devices available in the literature to allow the reader a better comprehension of the effects of these devices on the treated segment and on the adjacent segments of the spine. For this reason, our analysis will be limited to the interspinous devices that have biomechanical studies published in the literature.

## 2. Classification of Interspinous Devices

The interspinous devices currently in the market could be classified into two main groups: motion preservation devices and devices that fuse the interspinous space. 

*(i) Motion Preservation Devices.* They may be further subdivided into devices that oppose the extension in a rigid manner and devices that oppose it in a flexible manner. Rigid, or static, devices consist of noncompressible materials (metal, synthetic polymers, etc.). Although they display very different biomechanical properties, these devices have the same mechanism of action: they provide a wedge between the spinous processes to ensure a consistent level of distraction during extension. Although the biomechanical characteristics of flexible/dynamic devices are very different due to their material and to their shape, they offer a higher level of elasticity that allows their deformation during extension of the segment in which they have been implanted so they act as a rearshock absorber. While rigid devices may be compared to a stone preventing a door from opening, flexible devices may be compared to a rubber stopper.

*(ii) Fusion Devices.* This kind of devices ranges from paired plates with teeth to U-shaped devices with wings that are attached to the spinous process. They are intended to be an alternative to pedicle screws and rod constructs to aid in the stabilization of the spine with interbody fusion. For use in combination with other implants with the intent to fuse two adjacent spinal segments, it has been proposed that interspinous fixation systems are less invasive and present fewer risks than pedicle or facet screws.

## 3. Biomechanics Effects of Interspinous Devices

### 3.1. Biomechanics Effects of Nonfusion Interspinous Devices

From the review of the studies on the biomechanics of nonfusion interspinous devices available in the literature, we have focused our attention on the analysis of the following biomechanical effects:

1. influence on the range of movement (ROM) of the treated segment and of the adjacent segments,
2. influence on the size of the spinal canal area and foraminal canals area,
3. effects on the intradiscal pressure, disc load, and facet load,
4. influence on the segmental tilt and instantaneous axis of rotation (IAR) of the treated segment.

#### 3.1.1. Influence on the Range of Movement (ROM) of the Treated Segment and of the Adjacent Segments

Lindsey et al. conducted a cadaver study to assess the effect of the X-Stop interspinous implant on the kinematics of the lumbar spine at the instrumented and adjacent levels [7]. They observed that, at the implanted level, ROM was significantly reduced in flexion-extension, while the other directions were not affected. The results of this study also showed that the kinematics of the adjacent levels during flexion-extension, axial rotation, and lateral bending were not significantly affected. They also showed that the sagittal angle was affected by the device implantation; they demonstrated a 2° decrease in lordosis (more flexed kyphotic position) from L2 to L5, having no effect on the kinematics at the adjacent segments.

Phillips et al. in 2006 published a cadaveric study about the effects of the DIAM device on the biomechanical response of the lumbar spine in flexion-extension, lateral bending, and axial rotation after partial facetectomy and discectomy [8]. Insertion of the DIAM device after discectomy restored the angular motion below the level of the intact segment in flexion-extension. In lateral bending, DIAM device insertion reduced the increased motion induced by discectomy, but not to the level of the intact segment. The DIAM device insertion did not reduce the increased axial rotation induced by discectomy, and the axial rotation remained larger than the intact value.

In a biomechanical* in vitro* study, the stabilisation effect of the Coflex device was tested in partially and completely destabilized segments and it was compared to the use of pedicle screw [9]. The results for flexion/extension and axial rotation suggest that the Coflex device would be clinically useful in these two planes. It allows motion that is significantly less than the motion found in the partially destabilized and completely destabilized specimens and this motion is not significantly different from that shown by the intact specimens. The results in both flexion/extension and axial rotation illustrate that the device offers nonrigid fixation and has the ability to restore the destabilized specimen back to its normal motion characteristics in these two planes.

Lafage et al. in 2007 published a combined* in vitro* and finite-element analysis to assess the biomechanical effect of the Wallis device on the biomechanical behavior of a vertebral segment [10]. Intact segments, injured segments, and instrumented segments (L4-L5) were compared under load in flexion-extension, lateral bending, and torsion. The effect of the implant appeared mainly in flexion-extension: experimental results showed reduced range of motion of the instrumented spine regarding the injured and intact ones; and finite-element analysis indicated a decrease of disc stresses and increase of loads transmitted to the spinous processes.

Another cadaveric study about the same interspinous device published in 2010 showed that the Wallis device reduced flexion-extension at L3-4 by 13.8% but increased lateral bending and axial rotation ROM by 6.2 and 0.4%, respectively [11].

Wilke et al. in 2008 conducted an* in vitro* study to assess the biomechanical effect of four different interspinous implants (Coflex, Wallis, DIAM, and X-Stop) [4]. Twenty-four human lumbar spine specimens were divided into four equal groups and tested with pure moments in flexion/extension, lateral bending, and axial rotation: (1) intact, (2) defect, and (3) after implantation. Range of motion and the intradiscal pressure were determined. In general, the defect caused an increase in ROM compared to the intact condition in all loading directions.

Implantation of the Coflex, Wallis, DIAM, and X-Stop devices could not compensate this destabilizing effect in none of the three loading directions, except for extension. The implants Coflex, DIAM, and X-Stop allowed more flexion than in the intact state but with similar results to the defect state, while the Wallis implant tended to restabilize the specimens to the values of the intact specimens. In lateral bending generally, the implants Coflex, Wallis, DIAM, and X-Stop allowed slightly more motion compared to the intact state. In axial rotation generally, the implants were not able to compensate the destabilization caused by the defect.

In 2009 an* in vitro* biomechanical study was published to evaluate the effect of the In-Space interspinous spacer on the range of motion (ROM) and intervertebral disc pressure (DP) at the implanted level and at adjacent levels [12]. The extension ROM at the implanted level after the In-Space implant with or without discectomy was statistically significantly reduced. An increase of ROM at the adjacent levels compensated for the reduction at the implanted level. There was no statistically significant change in ROM in any of the other modes of motion at any of the levels studied. Likewise, the DP reduction at L3-4 during extension was statistically significant, but, in all other modes of motion, there was no statistically significant change in DP at any measured level. Authors concluded that the In-Space interspinous spacer both stabilizes the spine and reduces the intervertebral disc pressure at the instrumented level during extension without significative effects on the adjacent segment.

Hartmann et al. [13] in 2011 conducted an* in vitro* biomechanical evaluation on the changes in the range of motion of the affected and adjacent segments following implantation of 4 different interspinous devices: Aperius, In-Space, X-Stop, and Coflex. This study was focused on the evaluation of the effect of preload condition on range of motion of the lumbar spine implanted with these devices. All interspinous devices caused a significant reduction of the ROM in extension at the instrumented segment without significantly affecting the other directions of motion with and without application of preload. The ROM in flexion was reduced by all implants only when the preload was applied. All tested devices showed an increase in adjacent segment range of motion.

#### 3.1.2. Influence on the Size of the Spinal Canal Area (SCA) and Foraminal Canals Area (FCA)

Richards et al. quantified the effect of the X-Stop interspinous spacer on the dimensions of the spinal canal and neural foramina during flexion and extension [5]. Canal and foramina dimensions were compared between the intact and implanted specimens. In extension, the implant significantly increased the canal area by 18%, the subarticular diameter by 50%, the canal diameter by 10%, the foraminal area by 25% (from 106 to 133 mm^2^), and the foraminal width by 41%. This shows that X-Stop implant prevents narrowing of the spinal canal and neural foramina during extension.

Lee et al. reported that the cross-sectional foraminal area using the X-Stop device at the implanted level was increased by 36.5% (or 22 mm^2^) using MRI in ten elderly lumbar spinal stenosis patients [14]. The authors reported a mean expansion of the spinal canal after insertion of the X-Stop device of 22% with significant differences between the standing, the seated neutral, and the seated extended positions.

Siddiqui et al. also observed that the X-Stop device implantation enlarged the foraminal area in extension at a single diseased (with 20% increase at left side) and at two diseased levels (with 20–32% increase) in 26 elderly lumbar spinal stenosis patients using a positional MRI [15].

Wan et al. [16] measured the vertical (gap) and horizontal (lateral translation) shortest distances in the interspinous space at the implanted and adjacent segments during weight-bearing functional activities before and after X-Stop device implantation. The authors reported an increase of the foraminal area of 32.9% (or 32 mm^2^), of the foraminal width of 24.4% during extension, but with minimal change in standing and flexion. In their study, the authors demonstrated that the implantation of the X-Stop device in patients with symptomatic lumbar spine stenosis (LSS) provides an effective distraction of the interspinous space* in vivo* without causing significant kinematic disturbances at the adjacent segments.

A study published in 2012 evaluated the biomechanical effects of Aperius PercLID in 37 patients with lumbar spinal stenosis and claudicatio spinalis [17]. The authors reported a significative increase of foraminal cross-sectional area (from 125.91 preoperatively to 148.17 mm2 at last follow-up assessment). The mean increase was 21.55 mm2, corresponding to 17.60% of average foraminal area.

#### 3.1.3. Effects on the Intradiscal Pressure and Facet Load

Swanson et al. in 2003 conducted a cadaveric disc pressure study after the implantation of an “appropriate size” X-Stop device [18]. A pressure transducer measured intradiscal pressure and annular stresses during flexion, neutral, and extension positions. The authors reported a reduction of the pressures in the posterior annulus and nucleus pulposus by 63% and 41%, respectively, during extension and by 38% and 20%, respectively, in the neutral and standing positions without significant change of the intradiscal pressures at the adjacent levels as shown in the same study published by Lindsey et al. [7].

In a study published in 2005 by Wiseman et al., facet loading parameters of lumbar cadaver spines were measured during extension before and after placement of an interspinous process implant (X-Stop) [19]. At the implanted level, the mean peak pressure, average pressure, contact area, and force were significantly reduced without significative changes at the adjacent levels with the exception of contact area at the level above the implant. This suggests that use of an interspinous implant could cause adjacent level facet pain or accelerated facet joint degeneration.

In the study published by Wilke et al. in all four implant groups (Coflex, Wallis, DIAM, and X-Stop), the intradiscal pressure was strongly released in extension [4]. In all other loading directions, flexion, lateral bending, and axial rotation, none of the implants caused a significant change in the intradiscal pressure.

In 2009, a biomechanical study was published that investigated the effect of different degrees of distraction of interspinous processes on the lumbar intervertebral disc pressure distribution [20]. The authors hypothesized that, after placement of an interspinous device, different distraction degrees would cause different changes of disc pressure distribution at the level of instrumentation. The ideal implant may be the one which could significantly decrease the intradiscal pressure in the posterior annulus and in the nucleus and redirect a large portion of the load away from the intervertebral disc to the spinous processes in the extension and neutral positions, with no appreciable load change in other parts of the disc at the instrumented level.

The authors found a positive correlation between the spacer height and load sharing. It was found that an interspinous device with a spacer height equal to the distance of the interspinous process in the neutral position can share the biomechanical disc load without a significant change of load in the anterior annulus. After placement of the implant with a spacer height equal to the interspinous processes distance in the neutral position, about 46% of the load in the posterior annulus can be shared by the implant in extension. After placement of the IPD spacer height obviously higher than the spinous processes distance in the neutral position, the load of the posterior annulus could be significantly shared in extension, neutral, and flexion positions. The load of the anterior annulus is increased about 400% in these positions and this can accelerate degeneration of the disc. The interspinous devices act as a fulcrum in segment motion and redirect the force from the respective posterior annulus to the spinous process; the degree of distraction of the interspinous process caused by the “fulcrum” is correlated with load distribution on the intervertebral disc.

Lazaro et al. conducted an* in vitro* biomechanical study about the alteration of the normal biomechanics after insertion of an In-Space interspinous spacer by a nondestructive cadaveric flexibility testing [21]. After In-Space device insertion, the authors recorded a significative reduction of the range of motion during extension and a significative less reduction of the foraminal height during extension (compared with the normal state). The interspinous device reduced the mean facet load by 30% during flexion and 69% during extension. The lack of alteration in coupling also supports that kinematics did not change in other directions of loading.

#### 3.1.4. Influence on the Segmental Tilt and Instantaneous Axis of Rotation (IAR) of the Treated Segment

In the study published by Wilke, the creation of the standardized defect caused a slightly kyphotic deformation of the specimens. This kyphosis ranged between 0.5° and 0.7° in median compared to the intact state (0°). After implantation of the 4 interspinous devices analysed (Coflex, Wallis, DIAM. and X-Stop), this segmental tilt became different between the four implant groups. While implantation of the DIAM device caused an increasing kyphosis, implantation of the Wallis or X-Stop device had almost no effect on the kyphosis caused by the defect. This is valid also for the Coflex device; however, in this group, the range of the single values was larger.

In a biomechanical study [22] on a lumbar porcine model, Anasetti et al. tested the effects of the use of two different sizes of DIAM device (10 mm and 14 mm) placed in 2 different positions: one representing a standard placement and the other a more anterior placement. The authors found that the DIAM device implantation induced a shift toward kyphosis at the implanted level. Generally, all devices sizes and positions led to a shift of the ICR paths toward the posterior direction, in both flexion and extension. Without the laces, the ICRs in flexion approached those of the intact spine segment, going toward the center of the disc. This result is very likely due to the less significant kinematic role of the device in flexion when implanted without the laces. With regard to the influence of the position and of the size of the device, the 14 mm device is oversized in the anterior position, thus leading to a high flexed neutral position. Contrastingly, the 10 mm device had a limited effect in the anterior position on the neutral position and thus on the flexion motion. The 14 mm device in both positions led to more limited movements of the ICRs during flexion and extension. This result would probably imply a more pronounced pivot role of the larger sized device during motion as compared with the smaller one.

### 3.2. Biomechanics Effects of Fusion Interspinous Devices 

The introduction in the market of fusion interspinous devices is relatively recent so there are less studies regarding the biomechanical effects of these devices mainly focused on the ROM reduction compared to pedicle screws constructs.

Kettler et al. performed a biomechanical* in vitro* study on a different version of the Coflex interspinous implant, called Coflex Rivet, in which the device is screw-fixed to the spinous processes [23]. The new device was tested for flexibility and load transfer and, unlike the original Coflex implant, it is shown to increase stability only in extension as described in other biomechanical studies. Compared to the defect condition (bilateral hemifacetectomy with resection of the flaval ligaments), both implants had a strong stabilising effect in extension. Also Coflex Rivet strongly stabilized in flexion and was able to compensate the destabilising effect of the defect in axial rotation and lateral bending. The authors believed that the biomechanical characteristics of this new implant might even make it suitable as an adjunct to fusion, which would be a new indication for this type of device.

Wang et al. conducted a biomechanical study on the CD HORIZON SPIRE fixation system [24]. The authors compared the stability provided by the SPIRE with unilateral and bilateral pedicle screw system in destabilized spines with or without anterior allograft support. Used alone, or in conjunction with an interbody cage, the SPIRE provided a great stability in flexion and extension and the limitation of motion appears to be equal to bilateral pedicle screw system. In lateral bending and axial rotation, the SPIRE had a less stabilizing effect and it reduced motion equal to unilateral pedicle screw system.

In the recent biomechanical study conducted by Karahalios et al. [25], the ASPEN device was compared with other devices standing alone and in conjunction with anterior lumbar interbody fusion (ALIF) procedure. The authors found that the stand-alone ASPEN device decreased significantly the ROM in extension and flexion with less effects on the ROM in lateral bending and axial rotation. The use of ASPEN device and ALIF had a stabilization effect immobilized equal to ALIF and pedicle screw system and superior to ALIF and anterior plate system. The authors concluded that ASPEN device could be an alternative implant to pedicle screw system and anterior plate system when used in conjunction with ALIF. The use of the ASPEN device resulted in flexion at the index level, with a resultant increase in foraminal height. Compensatory extension at the adjacent levels prevented any significant change in overall sagittal balance.

Kaibara et al. [26] conducted a biomechanical study on ASPEN interspinous fixation device in combination with transforaminal lumbar interbody fusion (TLIF) and other posterior fixations in human cadaver spines. The use of the stand-alone ASPEN device significantly reduced motion in flexion and extension and the outcomes were similar to the effects obtained with the use of TLIF and bilateral pedicle screw system. In lateral bending and axial rotation, ASPEN device with and without TLIF showed inferior stability to bilateral pedicel screw. TLIF supplemented with ASPEN device and unilateral screw system provided equal stability as in TLIF with bilateral pedicle screws. The authors suggested the ASPEN device as a possible alternative to pedicle screw systems.

In 2013, Techy et al. [27] conducted a biomechanical study to evaluate the effect of the use ASPEN device as augmentation of an interbody cage or a pedicular screws fixation. After implantation of the ASPEN device to augment the interbody cage, there was a significant decrease in the ROM of 74% in flexion-extension (FE) but there was no significant change in lateral bending (LB) and axial rotation (AR). The construct with unilateral pedicle screws showed a significant reduction of FE by 77%, LB by 55%, and AR by 42% compared with control spine. The bilateral pedicle screws construct reduced FE by 77%, LB by 77%, and AR by 65% when compared with the control spine. The authors concluded that ASPEN device, which is used to augment an interbody cage, was able to provide FE stability comparable with the bilateral pedicle screw fixation. However, it provided minimal stability in LB and AR unless further augmented with pedicle screws.

Similar results were obtained by the study published by Gonzalez-Blohm et al. [28] in 2014. In this study, the authors evaluated the biomechanical performance of the ASPEN as a stand-alone device after lumbar decompression surgery and as supplemental fixation in a posterior lumbar interbody fusion (PLIF) construct. They suggested that the ASPEN device may be a suitable device to provide a flexion-extension balance after a unilateral laminotomy. PLIF constructs with ASPEN device and pedicle screws fixation performed equivalently in flexion-extension and axial rotation, but the PLIF-bilateral pedicle screws construct was more resistant to lateral bending. The authors recommended further biomechanical and clinical evidence to strongly support the use of this interspinous fusion device as stand-alone or as supplemental fixation to expandable posterior interbody cages.

## 4. Discussion and Conclusions

Biomechanically, all the different interspinous nonfusion devices, which are in the market today, increase stability in extension but are not able to compensate instability in axial rotation, in lateral bending, and in some cases in flexion [1, 3, 6]. Inserting a device between the spinous processes gives rise to a distractive effect at the affected site with an increase in the size of the spinal canal and foraminal canals, while adjacent levels that are unaffected by the device do not generally undergo appreciable influences [1, 23]. The presence of a rigid element acts as a fulcrum in extension movements, by attracting toward itself the axis of instantaneous rotation which is normally located in an anterior position near the facet joints during such movements, thus helping to relieve the load on the latter and on the rear part of the intervertebral disc; the degree of distraction of the interspinous process caused by the “fulcrum” is correlated with load distribution of the intervertebral disc [10]. Less studies analysed the influence of the implant size, placement, and fixation on the implanted segment and on the adjacent segments. The most appropriate implant size is still controversial. As suggested by the study conducted by Anasetti et al., the shift of the neutral position was related to the size and positioning of the device [22]. Small devices contributed to spine stabilization only to a limited extent, while too large devices could induce a kyphotic neutral position with the risk of disc overloading.

These concepts were also well documented by the biomechanical study published by Zheng et al. in which different sizes of the same device were evaluated [20]. The results of this study showed that the placement of an implant with the spacer height equal to the distance of the interspinous process was associated with a slight flexion of the segment and with a share of the load in the posterior annulus in extension with less effects on the dimension of the spinal canal and foramen. This implant size could be insufficient in the treatment of patients with lumbar spinal stenosis but it could be helpful in the treatment of patients with degenerative disc disease. However, the use of interspinous height higher than the interspinous processes distance is associated with a high degree of flexion of the segment and with a significative share of the load from the posterior annulus in extension, neutral, and flexion positions. The use of a big device is associated with a great increase of the dimension of the spinal canal and foramen; that is, it could be helpful in the treatment of patients with lumbar spinal stenosis. Unfortunately, the overdistraction of the interspinous process can accelerate disc degeneration by an excessive load on the anterior annulus. So the choice of the current implant size seems to be important for the patient clinical outcome (relief from pain in patients with lumbar spinal stenosis and prevention of the anterior disc degeneration). To achieve correct implantation and to avoid an overestimation of the device size, some authors recommend measuring the distance between the spinous processes or using the device templates, if provided, rather than the operating positions, which induce excessive spinal flexion.

**Figure 1:**
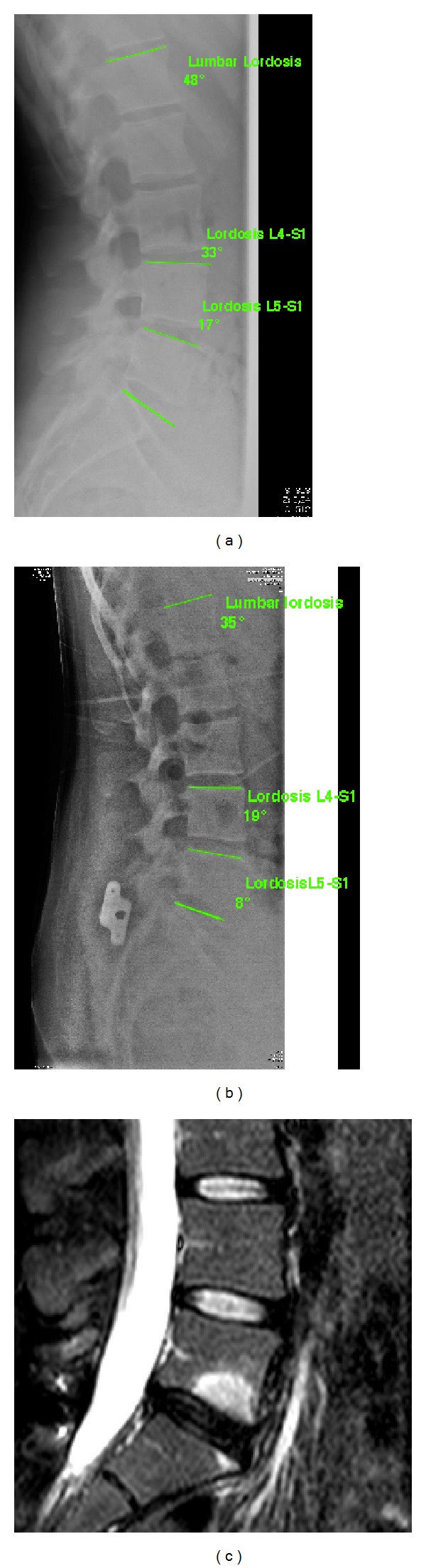
Segmentary L5-S1 kyphosis of after the implantation of a stand-alone ASPEN interspinous device with overload of the anterior part of the intervertebral disc. (a) Sagittal balance of lumbar spine before the implantation of the ASPEN device: lumbar lordosis 48°, L4-S1 lordosis 33°, and L5-S1 lordosis 17°. (b) Sagittal balance of lumbar spine after 4 years from the ASPEN device implantation: lumbar lordosis 35°, L4-S1 lordosis 19°, and L5-S1 lordosis 8°. (c) MRI scan after 4 years from the Aspen implantation that shows anterior disc endplate degeneration (Modic II).

The fusion devices encourage rigid stabilization of the level through the use of a spacer body that brings about controlled distraction and allows rigid stabilization through synthesis and fusion. These devices may also be used in stand-alone mode or with cages or other intersomatic devices to achieve complete fusion biomechanically equivalent to other more invasive fusion solutions [24–28]. From a biomechanical point of view, it is mandatory to consider that the interspinous device induces a segmentary kyphosis in a tract of the spine which is normally characterized by lordosis and it could cause overload of the anterior disk if used in the stand-alone configuration (Figure 1). Over time, if the interspinous device is used in combination with cage, this focal kyphosis could also have a negative impact on the interbody fusion and bone graft. Further studies are required to investigate these aspects. However, while biomechanical studies indicate that interspinous fixation devices may be similar to pedicle screw-rod constructs in limiting the range of flexion-extension, they may be less effective for limiting axial rotation and lateral bending [24, 28].

While in the literature the biomechanical effects of interspinous devices are well described, further high evidence studies dealing with complication rate and cost-effectiveness analysis are required. The world of interspinous space surgery is versatile and it opens up a number of options for treating spinal condition. Possible beneficiaries include elderly patients with severe concomitant conditions, when conventional open surgery is contraindicated.

We cannot, however, underestimate the risk that overstretching the list of indications may lead inevitably to a proliferation of failure, which would diminish the interest and consensus over this topic.
